# Real-World Evidence of Long-Term Disease Control in HER2-Positive Metastatic Breast Cancer Patients Treated with a First-Line Combination of Trastuzumab and/or Pertuzumab Plus Chemotherapy

**DOI:** 10.3390/cancers17213532

**Published:** 2025-10-31

**Authors:** Loïc Chaigneau, Eva Lapp, Taha Jai, Erion Dobi, Berenger Martin, Elsa Curtit, Virginie Nerich

**Affiliations:** 1 CHU Besançon, Service d’Oncologie, F-25030 Besançon, France; e1dobi@chu-besancon.fr (E.D.); elsa.curtit@univ-fcomte.fr (E.C.); 2 CHU Besançon, Pôle Pharmacie, F-25030 Besançon, France; eva.lapp@edu.univ-fcomte.fr (E.L.); taha.jai@edu.univ-fcomte.fr (T.J.); v1nerich@chu-besancon.fr (V.N.); 3 CHU Besançon, Unité de Méthodologie uMETH, Inserm Centre Investigation Clinique 1431, F-25030 Besançon, France; bmartin@chu-besancon.fr; 4 Université Marie et Louis Pasteur, INSERM, EFS-BFC, UMR 1098, F-25030 Besançon, France

**Keywords:** HER2-positive receptor, long-term responder, metastatic breast cancer

## Abstract

**Simple Summary:**

This study examines women with HER2-positive metastatic breast cancer who were treated with a combination of trastuzumab and/or pertuzumab plus chemotherapy as their first treatment. Out of 280 patients, 48 (17.5%) maintained control of their disease for at least three years. Most were women with an average age of 56.7 years, and around 70% had newly diagnosed metastatic cancer. Nearly 90% of patients responded to the treatment, with a median response duration of 5.8 years and a median progression-free survival of 11.0 years. These findings suggest that some patients can achieve long-term disease control, raising questions about treatment intensification and the possibility of stopping treatment. Future research is needed to identify factors that predict long-lasting responses.

**Abstract:**

**Background and Method**: The overexpression of the human epidermal growth factor receptor 2 (HER2) in breast cancer is correlated with accelerated tumor progression and an unfavorable clinical outcome. Since the introduction of trastuzumab in 2002, the treatment of HER2-positive breast cancer has been revolutionized, leading to significant improvements in survival. This retrospective, multicenter study aimed to describe the characteristics of patients with HER2-positive metastatic breast cancer (MBC) who maintained disease control for a minimum of three years after first-line therapy with trastuzumab and/or pertuzumab combined with chemotherapy. **Results**: Among 280 eligible patients, 48 (17.5%) were classified as long-term responders. The study population primarily consisted of women with a median age of 56.7 years at diagnosis; de novo metastatic presentation was observed in approximately 70% of cases. An objective response rate of nearly 90% was observed, with a median duration of response of 5.8 years. Median progression-free survival was 11.0 years [95% CI: 6.6—not reached], and median overall survival was not reached [95% CI: 10.9—not reached]. Furthermore, about 15% of patients were able to discontinue systemic therapy without immediate disease progression. **Discussion and Conclusions**: These findings indicate the potential of achieving prolonged disease control in a subset of patients with HER2-positive MBC, raising questions about therapeutic intensification and potential treatment discontinuation strategies. This study underscores the need for future research to identify predictive factors of durable response and assess the feasibility of adaptive treatment strategies, including planned treatment discontinuation.

## 1. Introduction

Amplification of the HER2 oncogene in breast cancer has long been recognized as a driver of aggressive tumor behavior and reduced survival [1]. The clinical introduction of trastuzumab in 2002 represented a landmark advancement, fundamentally altering the prognosis for patients with HER2-positive disease [2]. Early clinical trials established that integrating trastuzumab into standard chemotherapeutic regimens significantly delayed disease progression (median: 7.4 vs. 4.6 months, *p* < 0.001; hazard ratio for progression: 0.51 [95% CI: 0.41–0.63]) and extended overall survival (median: 25.1 vs. 20.3 months, *p* = 0.046; hazard ratio for death: 0.80 [95% CI: 0.64–1.00]) in the metastatic setting [3].

At present, the cornerstone of first-line therapy for HER2-positive MBC remains the combination of anti-HER2 targeted agents and cytotoxic chemotherapy, with taxane-based regimens being the most commonly employed. The incorporation of pertuzumab alongside trastuzumab and docetaxel has emerged as the preferred standard of care, supported by robust evidence from the CLEOPATRA trial demonstrating superior progression-free survival (median: 18.7 vs. 12.4 months; HR: 0.68 [95% CI: 0.58–0.80]; *p* < 0.001) and overall survival (median: 56.5 vs. 40.8 months; HR: 0.68 [95% CI: 0.56–0.84]; *p* < 0.001) [4,5]. Subsequent analyses, including the phase IIIb PERUSE study, confirmed the efficacy of a dual HER2 blockade across various taxane backbones (docetaxel, paclitaxel, or nab-paclitaxel) [6], findings further validated by real-world clinical practice data [7,8,9,10,11,12,13,14,15,16,17].

Recent advances have also explored the role of cyclin-dependent kinase 4/6 inhibitors in this population. The phase III PATINA trial reported that the addition of palbociclib following induction chemotherapy yielded significant and clinically relevant improvements in progression-free survival among patients with hormone receptor-positive, HER2-positive MBC [18].

While most patients experience disease progression within the first two years of treatment, some maintain non-progressive disease for three years or longer and are considered long-term responders [16,17]. In this context, the primary aim of this real-world evidence study was to investigate the profile of long-term responders (patients with non-progressive disease for at least three years) receiving first-line treatment of HER2-positive MBC with a combination of trastuzumab and/or pertuzumab plus chemotherapy. The profile was investigated in terms of clinical, tumor, and treatment characteristics, as well as survival and safety. Secondary aims were to assess the long-term responder rate, treatment duration, rate and reasons for treatment discontinuation, time to disease progression, and, if applicable, time to subsequent systemic cancer therapy.

## 2. Materials and Methods

### 2.1. Study Design and Setting

A retrospective, multicenter (one university hospital and two general hospitals) study was conducted at the Institut Régional Fédératif du Cancer de Franche-Comté (France, Franche-Comté region, which has a population of approximately 1.2 million).

### 2.2. Population

**Figure 1:**
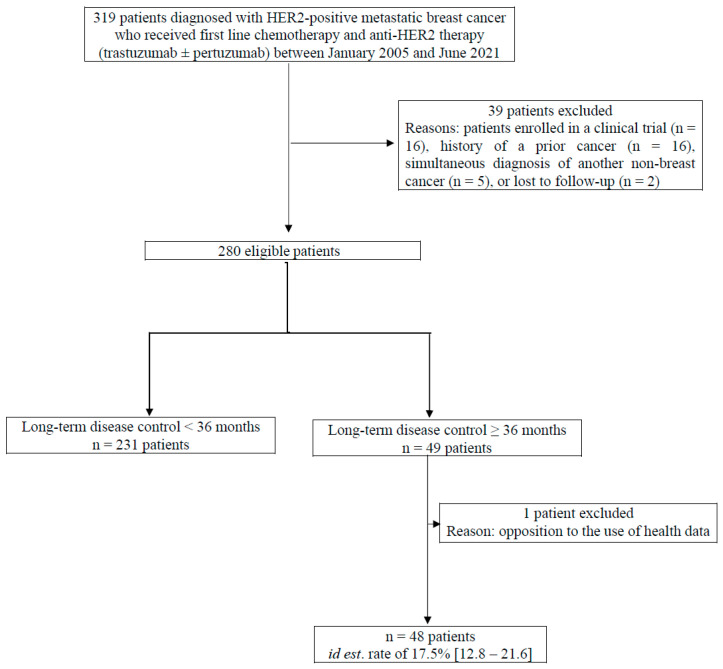
Flowchart of patient selection in the META-HEROES cohort. Legend: [] = 95% confidence interval.

From January 2005 to June 2021, all consecutive women aged 18 years or older with histologically or cytologically confirmed HER2-positive MBC who were naïve to previous systemic therapy for MBC, who received first-line treatment with a combination of trastuzumab and/or pertuzumab plus chemotherapy outside of a randomized controlled trial, and who had non-progressive disease for three years or longer were included in the META-HEROES cohort (Figure 1). Patients enrolled in a clinical trial, those with a history of prior cancer, those with a simultaneous diagnosis of another non-breast cancer, or those lost to follow-up were excluded.

Eligible patients were identified using BPC^®^ software (University Hospital, Besançon, France, version 23/01/25), a computerized regional physician order entry system capable of tracking intravenous and oral systemic cancer therapies according to tumor type. Age, clinical characteristics (medical history, World Health Organization (WHO) performance status), tumor characteristics (date of initial and metastatic diagnosis, stage, HER2 status, estrogen and progesterone receptor status (positive if at least 10% of cells tested have estrogen and/or progesterone receptors), visceral and non-visceral lesions), treatment (first-line systemic therapy for MBC, objective response (yes vs. no) and its duration, safety (grade 3–4 serious adverse events (SAEs), treatment discontinuation and associated reasons, and second- and third-line therapies), and survival outcomes (progression-free and overall survivals) were retrospectively collected in an electronic case report form from the medical record and BPC^®^ software.

All patients meeting the inclusion and exclusion criteria were consecutively included, regardless of their health status.

### 2.3. Response Assessment

The course of disease and response to treatment were evaluated by an oncologist prior to each treatment administration (every week or every three weeks depending on protocol frequency). Assessments were based on clinical examination and laboratory results. Imaging assessments (CT scan and isotope bone scan or PET scan) of treatment efficacy were performed by the radiology or nuclear medicine department according to metastatic site and were reviewed by the medical oncologist every three months.

### 2.4. Safety Assessment

The oncologist evaluated the safety of systemic cancer therapy at each visit. Left ventricular ejection fraction was measured by echocardiography or scintigraphy at treatment initiation and every three months during treatment.

Each grade 3–4 serious adverse event identified in the medical records of the included patients was coded according to the MedDRA^®^ (Medical Dictionary for Regulatory Activities, Worldgate Drive Herndon, VA 20170-6008, USA) classification and monitored until complete resolution or the end of the patient’s participation in the study [19,20].

### 2.5. Statistical Analysis

The objective response rate was defined as the proportion of patients with the best overall response of confirmed complete response or partial response, assessed by the oncologist according to RECIST v1.1. Response duration was measured from the date of the first documented objective response to the date of the first documented disease progression or death from any cause.

PFS was defined as the time from initiation of first-line systemic therapy for metastatic cancer to disease progression or to death from any cause or the last follow-up for survivors. OS was defined as the time from initiation of first-line systemic therapy for metastatic cancer to death from any cause or the last follow-up for survivors. Patients alive on 31 December 2024, the last date of analysis, were censored. Survival curves were estimated using the Kaplan–Meier method and medians were estimated with a 95% CI.

Continuous variables such as age and durations were described by mean ± standard deviation and median (range), qualitative variables by number and percentage, and rates (objective response rate, long-term responders, treatment discontinuation, and grade 3–4 serious adverse events) by percentage and a 95% CI. Statistical analyses were performed using SAS v9.4 (SAS Institute, Cary, NC, USA).

### 2.6. Registration

The study has been registered by the Clinical Research and Innovation Delegation of the University Hospital Center of Besançon under the number 2022/721. It was conducted in compliance with the reference methodology MR004 of the Commission Nationale de l’Informatique et des Libertés (CNIL, French data protection authority), which governs the processing of personal data for the purposes of studies, evaluations, or research not involving human participants. All subjects included received written information and were given sufficient time to express their opposition to the collection and processing of their data if they wished.

## 3. Results

### 3.1. Patient Population

From January 2005 to June 2021, 48 women out of 280 eligible patients who initiated first-line treatment remained progression-free after three years. The long-term disease control rate was estimated at 17.5% [95% CI: 12.8–21.6].

### 3.2. Demographic, Clinical, and Tumor Characteristics

More than two thirds of patients (*n* = 33) had de novo metastatic disease (Table 1). All 15 patients with early breast cancer at initial diagnosis had undergone breast surgery (8 tumorectomies and 7 mastectomies), and 14 received neoadjuvant or adjuvant chemotherapy. Among the 14 patients with HER2 overexpression, 9 received adjuvant trastuzumab, while the remaining 5 did not, as trastuzumab had not yet been approved for the treatment of HER2-positive early BC at that time. The median disease-free interval for these 15 patients was estimated at 5.4 years (1.4–20.3).

The mean age at metastatic diagnosis was 56.7 ± 12.5 years (range: 27.3–80.1), and 39 out of 48 (81%) patients were under 65 years old (Table 1). At the initiation of first-line systemic therapy for metastatic cancer, 79% of patients had a WHO performance status of 0 (*n* = 22) or 1 (*n* = 12). Estrogen receptor positivity was observed in more than 60% of patients, progesterone receptor positivity in 40%, and all patients had HER2-positive disease (46 with an immunohistochemistry score of 3+ and two confirmed by FISH). Nearly two thirds (*n* = 30) had visceral lesions, while nearly one third (*n* = 15) had bone-only metastases. Six patients (12%) received prior local therapy before initiating the combination of trastuzumab and/or pertuzumab plus chemotherapy.

### 3.3. Treatment Characteristics

All patients who received trastuzumab alone (*n* = 17, 35%) or in combination with pertuzumab (*n* = 31, 65%) also received taxane-based chemotherapy as follows: docetaxel alone (*n* = 31, 65%), docetaxel switched to paclitaxel due to treatment-related adverse events (*n* = 6, 12%), or paclitaxel alone (*n* = 11, 23%) (Table 2).

An objective response was observed in approximately 90% of cases, including 23 complete responses and 20 partial responses, while five patients had stable disease. The mean duration of response was estimated at 5.8 ± 3.4 years (range: 0.1–17.0). No statistically significant difference was observed between a single and dual anti-HER2 blockade (*p*-value > 0.05).

Systemic cancer therapy was discontinued in nearly half of the patients (*n* = 23), with most of them because of disease progression (*n* = 14). Other reasons for discontinuation included death (*n* = 2), adverse events (*n* = 2; grade <3, related to decreased left ventricular ejection fraction without disease progression at data cut-off), and medical or patient decision (*n* = 5, with two patients showing no disease progression at data cut-off, one showing progression three years later, and two who died 16 and 20 months later, respectively).

### 3.4. Grade 3–4 Serious Adverse Events

A grade 3–4 SAE was reported in seven patients who were in complete (*n* = 5) or partial (*n* = 2) response at their time of last administration of systematic cancer therapy. Treatment discontinuation occurred in three cases, with two due to disease progression (4.6 and 6.5 years later, respectively) and one due to medical decision (with no disease progression at the last follow-up).

Two systemic cancer therapies were imputed, namely docetaxel (*n* = 5) and paclitaxel (*n* = 2) (Table 3). Reported SAEs included blood and lymphatic system disorders (febrile neutropenia, *n* = 2), nervous system disorders (peripheral neuropathy, *n* = 2), gastrointestinal disorders (enterocolitis, *n* = 1), immune system disorders (allergic reaction, *n* = 1), and respiratory, thoracic, and mediastinal disorders (allergic interstitial pneumonitis, *n* = 1).

### 3.5. Survival

**Figure 2:**
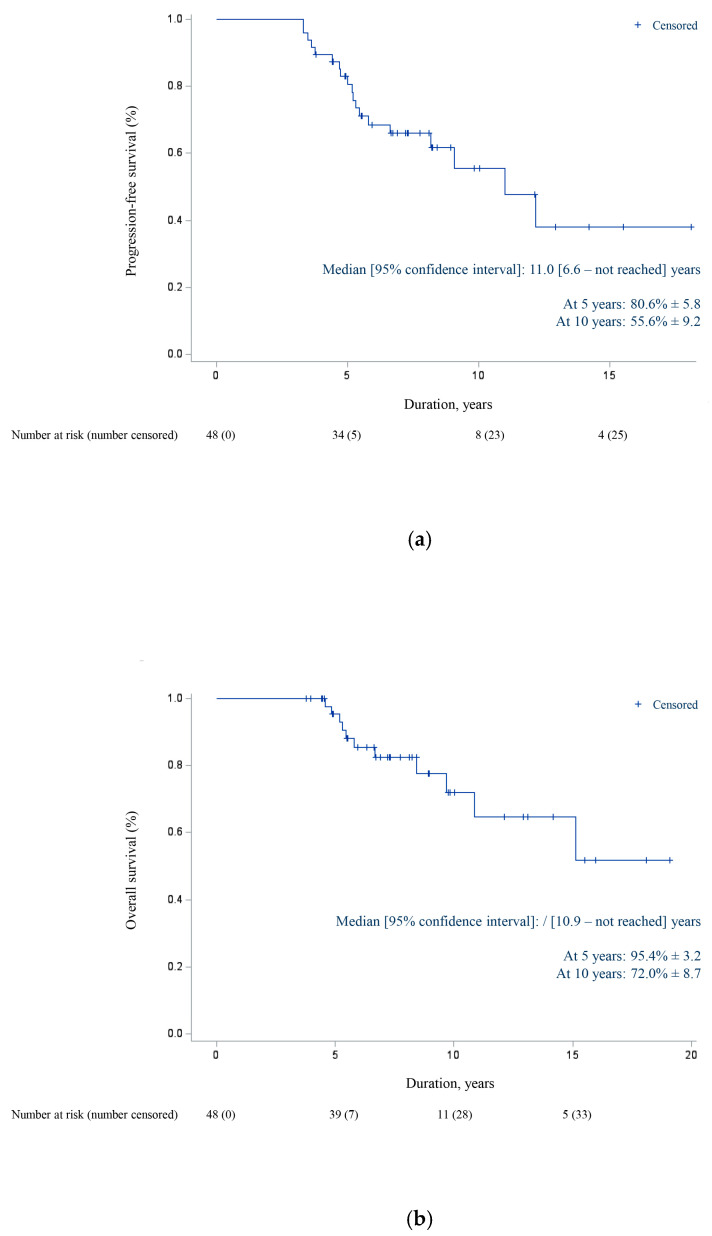
Kaplan–Meier estimates of (**a**) progression-free survival and (**b**) overall survival in first-line combination of trastuzumab and/or pertuzumab plus chemotherapy for HER2-positive metastatic breast cancer. Abbreviations: CI = confidence interval; % = percentage.

**Figure 3:**
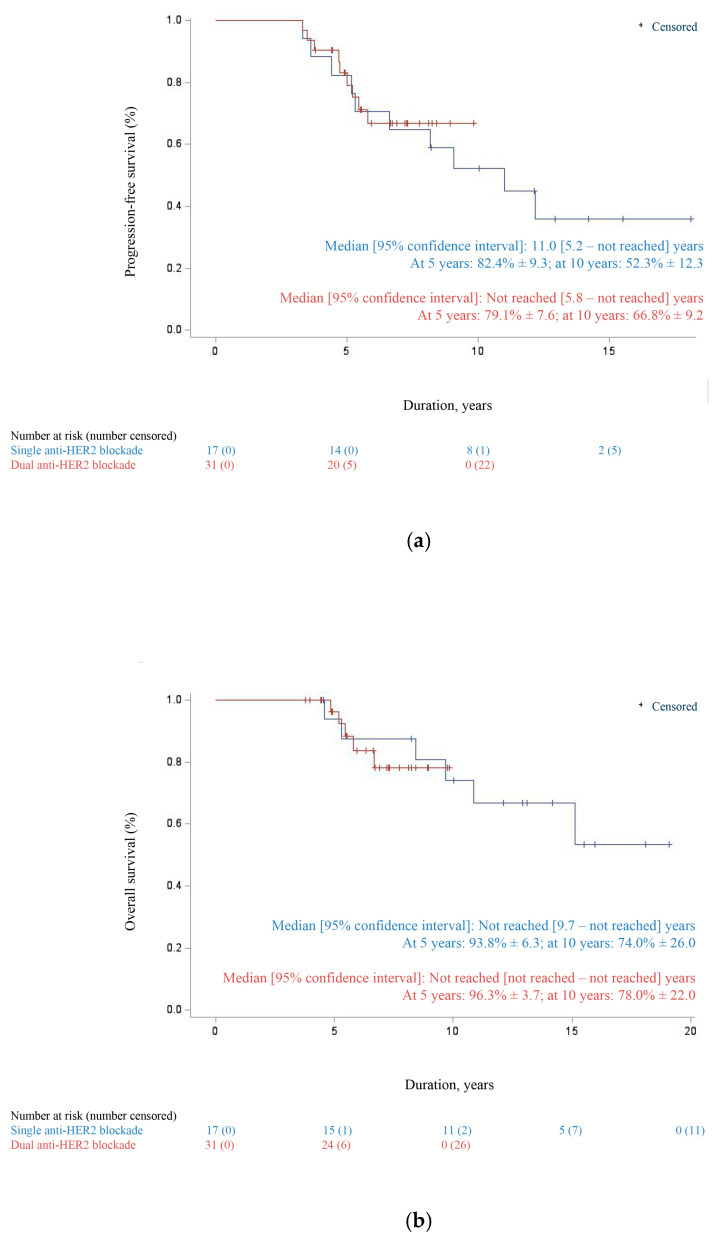
Kaplan–Meier estimates of (**a**) progression-free survival and (**b**) overall survival in first-line trastuzumab plus chemotherapy vs. combination of trastuzumab and pertuzumab plus chemotherapy for HER2-positive metastatic breast cancer. Abbreviations: CI = confidence interval; % = percentage.

As of 31 December 2024, the last date of analysis, the mean follow-up was 8.3 ± 3.8 years and the median follow-up 7.2 years (3.8–19.1). At that time, 19 patients had progressed with a median time to disease progression of 69.4 ± 30.6 months (39.5–145.9). Median PFS was 11.0 years [95% CI: 6.6—not reached] (Figure 2). The estimated Kaplan–Meier PFS rates were 80.6% ± 5.8 at 5 years and 55.6% ± 9.2 at 10 years. No statistically significant difference was observed between the single and dual anti-HER2 blockade (*p*-value > 0.05) (Figure 3).

Eleven patients had died at the time of the analysis. Median OS was not reached [95% CI: 10.9—not reached] (Figure 2). The estimated Kaplan–Meier OS rates were 95.4% ± 3.2 at 5 years and 72.0% ± 8.7 at 10 years.

### 3.6. Subsequent Systemic Cancer Therapy

**Figure 4:**
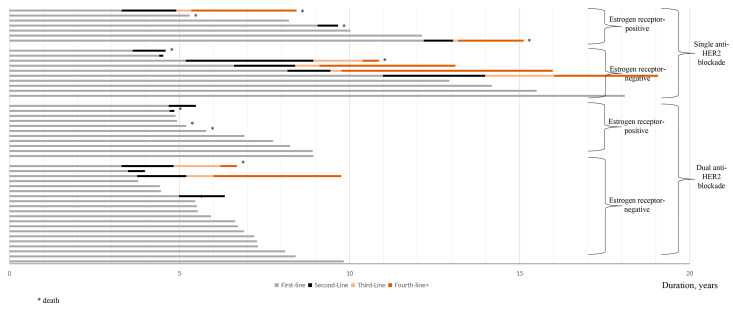
Details of the different systemic cancer therapies.

All 14 patients who progressed after first-line treatment with a combination of trastuzumab and/or pertuzumab plus chemotherapy for HER2-positive MBC received a second-line systemic cancer therapy, involving trastuzumab emtansine (*n* = 6), trastuzumab deruxtecan (*n* = 4), or a combination of trastuzumab and/or pertuzumab with docetaxel (*n* = 4) (Table 4 and Figure 4). One patient progressed three years after discontinuing systemic cancer therapy due to a medical decision and received a combination of trastuzumab and pertuzumab plus taxane as second-line systemic cancer therapy. An objective response was observed in nearly 27% of cases (two complete responses and two partial responses), while six patients achieved stable disease and five were not evaluable.

The discontinuation of systemic cancer therapy occurred in nearly three quarters of cases (eight because of disease progression and three deaths). All eight patients who progressed after second-line systemic cancer therapy received a third-line systemic treatment.

## 4. Discussion

### 4.1. Prolonged Survival

Our findings reveal that approximately 17.5% of patients with HER2-positive metastatic breast cancer (MBC) who received first-line anti-HER2 therapy—combining trastuzumab and/or pertuzumab with a taxane—achieved durable disease control exceeding three years, with a median progression-free survival (PFS) of 11.0 years [95% CI: 6.6—not reached]. These data align with prior evidence from real-world registries (e.g., SONABRE) and exploratory analyses from pivotal trials such as CLEOPATRA and PERUSE, which report similar long-term responder rates [4,5,6,17,21].

The SONABRE registry updated its results in September 2025, comparing patients treated in two different periods (2008–2012 vs. 2013–2017) [21,22]. Among those diagnosed with de novo metastatic disease after 2013, the 5-year PFS rose to 24%, in contrast to 10% in the earlier cohort. Correspondingly, the 5-year overall survival (OS) improved from 28% to 51%.

The CLEOPATRA trial, a landmark phase III study, established the addition of pertuzumab to trastuzumab and docetaxel as the standard of care for untreated HER2-positive MBC [5]. Compared with trastuzumab plus docetaxel alone, the triplet regimen significantly extended both PFS (12.4 vs. 18.7 months) and OS (40.8 vs. 56.5 months).

Similarly, the PERUSE trial, a non-randomized phase IIIb study, assessed various chemotherapy backbones combined with a dual HER2 blockade. It reported no major differences in efficacy across taxane types, with a median PFS of 20.7 months [95% CI: 18.9–23.1] and a median OS of 65.3 months [95% CI: 60.9–70.9] [6].

### 4.2. Clinical Factors Associated with Durable Response

In the SONABRE dataset, 63 out of 244 patients attained complete clinical response after first-line therapy, with a median PFS of 74.3 months [95% CI: 57.3–not reached] and a 5-year PFS rate of 63% [95% CI: 48–75%] [21,22]. Median OS was not reached, and the 5-year OS was 85%. Multivariate analysis identified the three following independent predictors: age <65 years, single-organ metastasis, and de novo disease at presentation. In our study cohort, the average age at metastatic diagnosis was 56.7 years (range: 27.3–80.1), with 81% under the age of 65. Nearly half (48%) had a single metastatic site, most frequently the bone.

De novo MBC represented around 70% of cases, in line with SONABRE and findings from Yeo Wong et al., who analyzed outcomes in 483 patients with de novo HER2-positive MBC treated between 1998 and 2015 [21,22,23]. In this group, median OS was 5.5 years [95% CI: 4.8–6.2], with 5- and 10-year OS rates of 54% and 18%, respectively. PFS remained stable at around 41% at both timepoints.

A noteworthy subgroup, classified as having no evidence of disease (NED) after multimodal therapy, demonstrated exceptional outcomes. Among them, 63 patients (13%) achieved complete radiologic remission, with 5-year PFS and OS rates of 100% and 98%, respectively. In contrast, non-NED patients had significantly worse outcomes, with a 5-year PFS of 12% and a 10-year OS of just 4%. NED patients more commonly had single-site metastases (79% vs. 51%, *p* = 0.005) and were more likely to undergo primary tumor surgery (59% vs. 22–25%, *p* ≤ 0.001).

Multivariate modeling confirmed that NED status (HR = 0.014, *p* = 0.0002) and hormone receptor positivity (HR = 0.72, *p* = 0.04) were independently associated with prolonged survival. Additionally, good performance status (PS 0–1 in 79%) and estrogen receptor positivity (60%) in our cohort further supported alignment with favorable prognostic indicators identified in the literature.

### 4.3. Therapeutic Intensification

In our series, approximately two thirds of patients received dual HER2-targeted therapy with pertuzumab and trastuzumab, consistent with CLEOPATRA trial recommendations [24]. All patients were treated with a taxane backbone, reinforcing the role of treatment intensification in achieving deep and sustained responses.

Recent trials are exploring ways to build on this standard. The phase III DESTINY-Breast09 trial compared trastuzumab deruxtecan plus pertuzumab versus standard regimens, reporting a median PFS of 40.7 months in the experimental arm vs. 26.9 months in the control arm. This was accompanied by higher complete response rates.

In parallel, the PATINA trial investigated the addition of the CDK4/6 inhibitor palbociclib to HER2-targeted maintenance therapy with endocrine therapy in hormone receptor-positive MBC, resulting in a PFS extension of over 15 months (44.3 vs. 29.1 months).

### 4.4. Durable Complete Responses and Treatment Discontinuation

Notably, nearly 90% of patients achieved an objective response, including 48% with complete response, and about 15% were able to discontinue systemic therapy without immediate progression. This observation, although rarely reported in published clinical trials, mirrors findings from Yeo Wong et al., where a subset (~13%) achieved undetectable disease, with 10-year survival rates exceeding 95% [22]. These data raise at least two key questions.

First, could therapeutic breaks be considered in highly selected patients—an approach not yet standardized but promising for improving quality of life and reducing long-term toxicities, especially cardiotoxicity? A group of experts published recommendations in 2024 on the management of these patients, raising the question of whether to stop or modify anti-HER2 treatment [25]. It is written “we would recommend that treatment be continued indefinitely, especially in those without complete response to therapy, provided they agree, until the development of progressive disease or unacceptable toxicity” and a little later “These results support the notion that after 5 years, clinicians could potentially consider discontinuing treatment in patients with a complete response to therapy on a case-by-case basis if this is something the patient desires”. The STOP HER2 (NCT05721248) and HEROES (French trial not registered on ClinicalTrials.gov) trials are exploring the potential of circulating tumor DNA (ctDNA) to revolutionize the management of HER2-positive cancers. In STOP HER2, ctDNA monitoring could enable the early detection of molecular residual disease and guide the safe discontinuation of anti-HER2 therapy in exceptional responders, potentially reducing unnecessary toxicity while maintaining efficacy. Similarly, HEROES might demonstrate that ctDNA dynamics can predict response and resistance to HER2-targeted therapies, allowing for real-time, personalized treatment adjustments. If validated, these approaches could establish ctDNA as a cornerstone for non-invasive, longitudinal disease surveillance and therapeutic decision-making in precision oncology. However, the final results are still awaited to confirm these hypotheses.

Second, this challenges the paradigm of incurability in MBC: could this dogma shift, just as it recently did in colorectal cancer [26,27]?

Currently, there is no validated predictive factor or guideline to initiate treatment discontinuation. However, while observing toxicity and quality of life in selected patients, a “stop-and-follow” strategy could be considered.

### 4.5. Limitations and Future Directions

The main limitations of our study are its retrospective nature and the limited size of the long-term responder subgroup. However, the extended period of follow-up data (up to 18 years) and the diversity of treatment strategies offer valuable insights complementary to randomized clinical trial data. Future prospective studies should aim to better identify biological predictors of prolonged response and assess the feasibility of adaptive treatment strategies, including planned treatment discontinuation, in patients with sustained complete remission.

## 5. Conclusions

In this study, we highlighted that a subset of patients treated with first-line anti-HER2 therapy achieved prolonged disease control beyond three years, with a median PFS exceeding 10 years in patients with metastatic HER2-positive breast cancer. Notably, this group was characterized by age under 65 years (for 81% of cases), approximately 70% of patients with de novo metastatic disease, limited bone metastases, and nearly 50% of patients with a complete response. Although no predictive factor or guideline is currently available to guide treatment discontinuation, the observation of toxicity and quality of life may lead to exploring a “stop-and-follow” strategy.

## Figures and Tables

**Table 1:** Clinical and tumor characteristics of 48 long-term responders treated with a first-line combination of trastuzumab and/or pertuzumab plus chemotherapy for HER2-positive metastatic breast cancer.

Number of Patients, *n* (%)	48 (100.0)
At metastatic diagnosis	
Age, years	
Mean ± standard deviation	56.7 ± 12.5
Median (range) Age <65 years	57.0 (27.3–80.1) 39 (81.3)
De novo metastatic disease	33 (68.8)
HER2 status *, *n* (%)	
Immunochemistry score 3+	46 (95.8)
Immunochemistry score 2+ (FISH-positive)	2 (4.2)
Estrogen receptor-positive **, *n* (%)	30 (62.5)
Progesterone receptor-positive **, *n* (%)	21 (43.8)
Surgery ± radiotherapy before combination of trastuzumab and/or pertuzumab plus chemotherapy ***	6 (12.5)
At initiation of combination of trastuzumab and/or pertuzumab plus chemotherapy	
WHO performance status, *n* (%)	
0	22 (45.8)
1	16 (33.3)
2	9 (18.8)
4	1 (2.1)
Metastasis biopsy, *n* (%)	21 (43.8)
HER2 status *, *n* (%)	
Immunochemistry score 3+	18 (85.7)
Immunochemistry score 2+	3 (14.3)
FISH-positive	2 (9.5)
Estrogen receptor-positive *, *n* (%)	15 (71.4)
Progesterone receptor-positive *, *n* (%)	11 (52.4)
Visceral or non-visceral lesions	
Visceral disease	30 (62.5)
Non-visceral disease (bone, skin and lymph node mestatases)	18 (37.5)
Bone only	15 (31.2)

Abbreviations: FISH = fluorescent in situ hybridization; HER2 = human epidermal growth factor receptor-2; *n* = number; WHO = World Health Organization; % = percentage. Legend: * if required, no difference between the initial and metastatic diagnosis; ** if at least 10% of cells tested have estrogen and/or progesterone receptors; *** 1 mastectomy; 3 metastasis surgeries (bone, hepatic, and lung), with 1 of them associated with 1 tumorectomy and 1 radiotherapy of metastases and 1 of them associated with 1 radiotherapy of metastases; 2 radiotherapy of metastases.

**Table 2:** Treatment characteristics of 48 long-term responders treated with a first-line combination of trastuzumab and/or pertuzumab plus chemotherapy for HER2-positive metastatic breast cancer.

Number of Patients, *n* (%)	48 (100.0)
Systemic cancer therapy, n (%)	
Chemotherapy	48 (100.0)
Docetaxel	37 (77.1)
75 or 100 mg/m^2^	26 (70.3)/11 (29.7)
Dose adjustment during treatment at least one time	17 (46.0)
Docetaxel replaced by paclitaxel	6 (16.2)
Paclitaxel (weekly protocol)	17 (35.4)
80 or 90 or 100 mg/m^2^	2 (11.8)/14 (82.3)/1 (5.9)
Dose adjustment during treatment at least one time	5 (29.4)
Anti-HER2 treatment	48 (100.0)
Trastuzumab	17 (35.4)
Trastuzumab plus pertuzumab	31 (64.6)
Objective response, *n* (%)	43 (89.6)
Duration of response, years	
Mean ± standard deviation	5.8 ± 3.4
Median (range)	4.9 (0.1–17.0)
In the process of treatment, *n* (%)	25 (52.1)
Main reason for discontinuation, *n* (%)	23 (47.9)
Disease progression	14 (60.9)
Death	2 (8.7)
Adverse event	2 (8.7)
Medical decision	4 (17.4)
Patient decision	1 (4.3)
Progression or death	19 (37.5)
Time to disease progression or death, months	
Mean ± standard deviation	69.4 ± 30.6
Median (range)	62.1 (39.5–145.9)

Abbreviations: HER2 = human epidermal growth factor receptor-2; *n* = number; % = percentage.

**Table 3:** Seven grade 3–4 serious adverse events according to their encoding in MedDRA^®^ (Medical Dictionary for Regulatory Activities) occurring in patients treated with a first-line combination of trastuzumab and/or pertuzumab plus chemotherapy for HER2-positive metastatic breast cancer.

	Imputed Systemic Cancer Therapy	Serious Adverse Events		
		Grade		Outcome
		3	4	
Blood and lymphatic system disorders				
Febrile neutropenia	Docetaxel	0	2	Inpatient hospitalization or prolongation of existing hospitalization
Gastrointestinal disorders				
Enterocolitis	Docetaxel	1	0	Persistent or significant disability/incapacity
Immune system disorders				
Allergic reaction	Paclitaxel	1	0	Persistent or significant disability/incapacity
Nervous system disorders				
Peripheral neuropathy	Docetaxel	1	0	Persistent or significant disability/incapacity
Peripheral neuropathy	Paclitaxel	1	0	Persistent or significant disability/incapacity

**Table 4:** Subsequent systemic cancer therapy among 19 long-term responders treated with a first-line combination of trastuzumab and/or pertuzumab plus chemotherapy for HER2-positive metastatic breast cancer patients who experienced progression.

Subsequent Systemic Cancer Therapy, *n* (%)	15 (78.9)
First	15 (78.9)
Regimen, *n* (%)	
Trastuzumab emtansine	6 (40.0)
Trastuzumab deruxtecan	4 (26.8)
Combination of trastuzumab and pertuzumab plus docetaxel	3 (20.0)
Combination of trastuzumab and pertuzumab plus paclitaxel	1 (6.7)
Combination of trastuzumab plus docetaxel	1 (6.7)
Objective response, *n* (%)	4 (26.6)
In the process of treatment, *n* (%)	4 (26.7)
Main reason for treatment discontinuation, *n* (%)	
Disease progression	8 (53.3)
Death	3 (20.0)
Second	8 (34.8)
Regimen, *n* (%)	
Trastuzumab emtansine	4 (50.0)
Trastuzumab deruxtecan	2 (25.0)
Trastuzumab plus capecitabine	1 (12.5)
Trastuzumab plus paclitaxel	1 (12.5)
Best overall response, *n* (%)	
Partial response	2 (25.0)
Stable disease	2 (25.0)
Progressive disease	4 (50.0)
In the process of treatment, *n* (%)	0 (0.0)
Main reason for treatment discontinuation, *n* (%)	
Disease progression	8 (100.0)

Abbreviations: HER2 = human epidermal growth factor receptor-2; *n* = number; % = percentage.

## Data Availability

All data generated or analyzed during this study are included in this article. Further enquiries can be directed to the corresponding author.
